# DNA Base Excision Repair in Plants: An Unfolding Story With Familiar and Novel Characters

**DOI:** 10.3389/fpls.2019.01055

**Published:** 2019-08-30

**Authors:** Teresa Roldán-Arjona, Rafael R. Ariza, Dolores Córdoba-Cañero

**Affiliations:** ^1^Maimónides Biomedical Research Institute of Córdoba (IMIBIC), Córdoba, Spain; ^2^Department of Genetics, University of Córdoba, Córdoba, Spain; ^3^Reina Sofia University Hospital, Córdoba, Spain

**Keywords:** DNA repair, DNA damage, DNA glycosylase, AP endonuclease, *Arabidopsis*

## Abstract

Base excision repair (BER) is a critical genome defense pathway that deals with a broad range of non-voluminous DNA lesions induced by endogenous or exogenous genotoxic agents. BER is a complex process initiated by the excision of the damaged base, proceeds through a sequence of reactions that generate various DNA intermediates, and culminates with restoration of the original DNA structure. BER has been extensively studied in microbial and animal systems, but knowledge in plants has lagged behind until recently. Results obtained so far indicate that plants share many BER factors with other organisms, but also possess some unique features and combinations. Plant BER plays an important role in preserving genome integrity through removal of damaged bases. However, it performs additional important functions, such as the replacement of the naturally modified base 5-methylcytosine with cytosine in a plant-specific pathway for active DNA demethylation.

## Introduction

The genomes of all organisms are susceptible to a variety of DNA lesions arising from endogenous and exogenous sources (Lindahl, 1993). Such threats to genome integrity are counteracted by diverse DNA repair pathways that are best understood in bacteria, yeast, and mammals. The base excision repair (BER) pathway is a critical DNA repair mechanism for removal of damaged bases arising from oxidation, alkylation, or deamination (Krokan and Bjoras, 2013). BER is initiated by DNA glycosylases that excise the damaged base and completed by additional proteins that remove the remaining sugar–phosphate moiety, fill the subsequent gap, and perform ligation. Knowledge about the BER pathway in plants has greatly advanced in the last two decades, mainly through studies in the model organism *Arabidopsis thaliana*, although additional progress has been made in other species. Results obtained so far indicate that plants have orthologs of most BER genes previously identified in other organisms. However, they also possess some plant-specific BER proteins, as well as distinctive enzyme combinations not found in other kingdoms. In the following sections, we first present a brief overview of the major stages in the BER pathway and then focus on the plant enzymes involved in every step, discussing their similarities and differences with BER factors from bacteria, yeast, and mammals.

## Overview of Base Excision Repair

**Figure 1:**
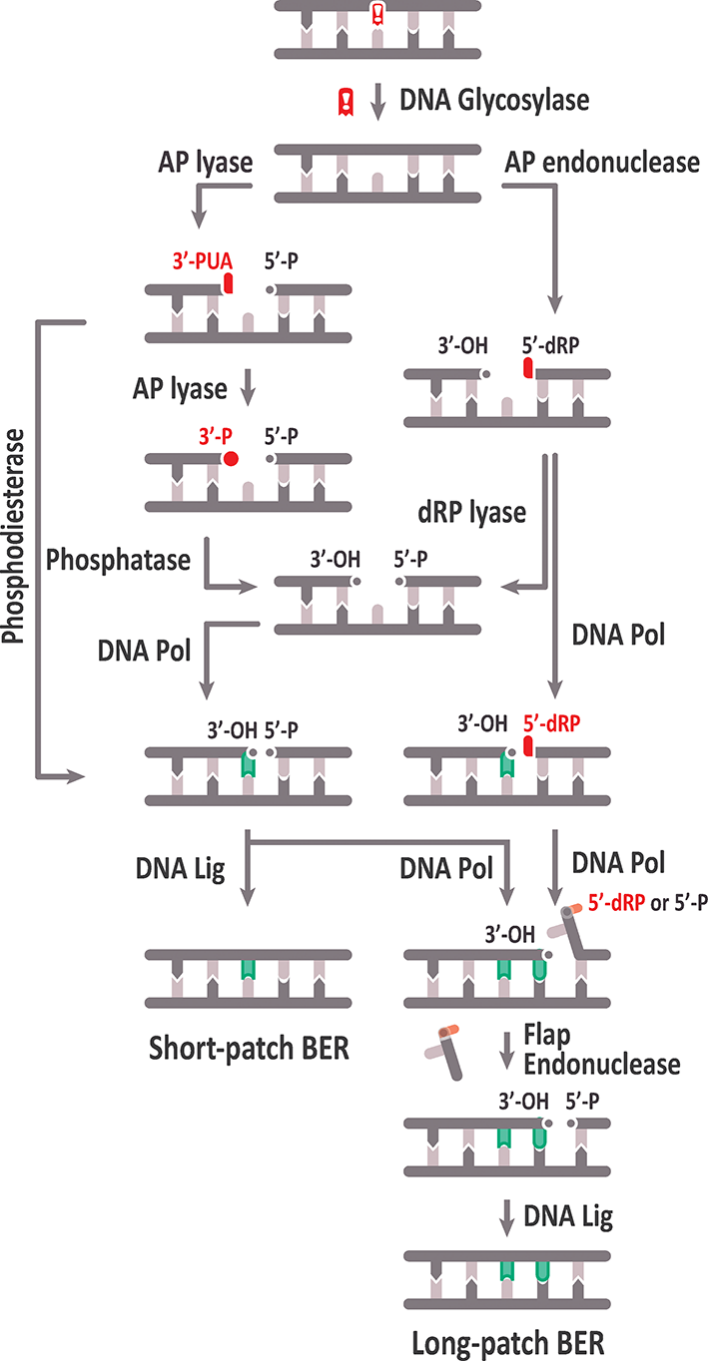
Schematic representation of the base excision repair (BER) pathway. See text for details.

BER is a complex mechanism that occurs in several steps: i) excision of the damaged DNA base, ii) cleavage of the sugar–phosphate backbone at the generated abasic (apurinic/apyrimidinic, AP) site, iii) clean-up of the resulting DNA ends, iv) gap filling through DNA synthesis, and v) DNA ligation (**Figure 1**). Repair factors involved in these stages have been identified primarily through studies in bacterial and mammalian systems.

The first BER step involves the excision of a modified or incorrect base through the action of a DNA glycosylase that cleaves the N-glycosidic bond, thus releasing the target base and leaving an AP site with the sugar–phosphate backbone intact. There are multiple DNA glycosylases with different substrates specificities (Friedberg et al., 2006; Jacobs and Schar, 2012).

Subsequent AP site processing can be achieved either by an AP lyase activity, usually associated with a subset of DNA glycosylases, or by AP endonucleases. Based on their catalytic activities, DNA glycosylases are classified into monofunctional and bifunctional. Monofunctional DNA glycosylases only remove the target base, thus generating an AP site, whereas bifunctional glycosylases possess an associated AP lyase activity that, after base excision, catalyzes 3′ incision to the AP site by β-elimination, generating 3′-α, β unsaturated aldehyde (3′-PUA), and 5′-hydroxyl (OH) termini. Some bifunctional DNA glycosylases perform a later δ-elimination reaction converting the 3′-PUA end in a 3′-phosphate (3′-P) terminus. The AP site generated by monofunctional DNA glycosylases is usually processed by an AP endonuclease, which cleaves the DNA backbone 5′ to the abasic site, thus generating 3′-OH and 5′‐deoxyribose-5-phosphate (5′-dRP) termini (Levin and Demple, 1990; Dianov et al., 1992).

Unconventional ends generated by AP lyases (3′-PUA or 3′-P) and AP endonucleases (5′-dRP) need to be processed to conventional 3′-OH and 5′-P termini, respectively, to allow DNA polymerization and ligation. Cleaning of 3′-PUA ends is performed by the 3′-phosphodiesterase activity of AP endonucleases, whereas the 3′-P termini are processed by a DNA 3′-phosphatase, which in mammalian cells is polynucleotide kinase phosphatase (PNKP) (Pascucci et al., 2002; Wiederhold et al., 2004). The 5′-dRP end must be processed to a 5′-P end by a deoxyribosephosphate (dRP) lyase activity that, in mammalian cells, is associated to DNA polymerase β (Srivastava et al., 1998).

Once the blocked termini have been processed to 5′-P and 3′-OH ends, gap filling may proceed either by insertion of one nucleotide (short‐patch or “single-nucleotide BER,” SP-BER) or 2–13 nucleotides (long-patch, LP-BER). In mammals, DNA polymerase β is involved in nucleotide insertion during SP-BER (Srivastava et al., 1998), and the resulting nick is ligated by a complex of XRCC1 and LigIIIα (Nash et al., 1997). In contrast, LP-BER requires replicative DNA polymerases (Pol δ and Pol ε, in mammals), which displace the strand containing the 5′-dRP terminus, generating a flap structure that is processed by a flap endonuclease (FEN1), and finally, the generated nick is sealed by LIG1 (Levin et al., 1997).

Plants possess homologs of most BER proteins identified in other organisms (Britt, 2002; Hays, 2002; Roldan-Arjona and Ariza, 2009b) (**Table 1**), and the complete BER pathway was reproduced *in vitro* using *Arabidopsis* cell extracts (Cordoba-Cañero et al., 2009). However, some factors are absent in plants, such as Pol β (Garcia-Diaz and Bebenek, 2007; Roy et al., 2008), others are encoded by multiple gene copies in plant genomes, such as PCNA and FEN1 (Kimura et al., 2003; Strzalka and Ziemienowicz, 2011), and additionally some BER proteins appear to be restricted to plants (Choi et al., 2002; Gong et al., 2002). Such differences suggest that plant-specific characteristics arose during BER evolution. In the following sections, we review plant factors involved in the main BER stages.

**Table 1:** Proteins involved in BER in bacteria, yeast, humans, and *Arabidopsis*.

BER enzyme	*E. coli*	*S. cerevisiae*	*H. sapiens*	*Arabidopsis*		
				Name	Gene ID	Reference
**DNA glycosylases**						
Uracil DNA glycosylases superfamily	Ung	Ung1p	UNG	AtUNG	AT3G18630	(Cordoba-Cañero et al., 2010)
	Mug		TDG			
			Smug1			
AAG			MPG	AthAAG	AT3G12040	(Santerre and Britt, 1994)
H2TH superfamily	MutM			AtFPG	AT1G52500	(Ohtsubo et al., 1998)
	Nei		NEIL1			
			NEIL2			
			NEIL3			
HhH-GPD superfamily	Nth	Ntg1p	NTHL1	AtNTH1	AT2G31450	(Roldan-Arjona et al., 2000)
		Ntg2p		AtNTH2	AT1G05900	(Gutman and Niyogi, 2009)
		Ogg1p	OGG1	AtOGG1	AT1G21710	(Dany and Tissier, 2001; Garcia-Ortiz et al., 2001)
	MutY		MYH	AtMUTY	AT4G12740	
	AlkA	Mag1p		AtAlkA	Two putative homologs	
	Tag			AtTag	Nine putative homologs	
			MBD4	AtMBD4L	AT3G07930	(Ramiro-Merina et al., 2013)
DML family				ROS1	AT2G36490	(Gong et al., 2002)
				DME	AT5G04560	(Morales-Ruiz et al., 2006)
				DML2	AT3G10010	(Ortega-Galisteo et al., 2008)
				DML3	AT4G34060	(Ortega-Galisteo et al., 2008)
**AP endonucleases**						
Xth family	Xth	Apn2p	APE1	ARP	AT2G41460	(Cordoba-Cañero et al., 2011)
				AtAPE1L	AT3G48425	(Li et al., 2015)
			APE2	AtAPE2	AT4G36050	(Li et al., 2015)
Nfo family	Nfo	Apn1p				
**3′** **DNA phosphatases**						
		Tpp1p	PNKP	ZDP	AT3G14890	(Petrucco et al., 2002; Martinez-Macias et al., 2012)
**DNA polymerases**						
Family A	Pol I	Pol γ	Pol γ			
				AtPolIA	AT1G50840	(Trasvina-Arenas et al., 2018)
				AtPolIB	AT3G20540	(Trasvina-Arenas et al., 2018)
			Pol θ	AtPol θ	AT4G32700	(Inagaki et al., 2006)
Family B		Pol α	Pol α	AtPol α	AT1G67630	
		Pol δ	Pol δ	AtPol δ	AT2G42120	
		Pol ε	Pol ε	AtPol ε	AT1G08260	
Family X			Pol β			
		PolIV	Pol λ	AtPolλ	AT1G10520	(Amoroso et al., 2011; Roy et al., 2011)
**Flap endonucleases**		Rad27p	FEN1	AtFEN1	AT5G26680	(Zhang et al., 2016b)
**DNA ligases**						
NAD^+^-dependent	LigA					
ATP-dependent		Cdc9p	LIG1	AtLig1	AT1G08130	(Cordoba-Cañero et al., 2011)
			Lig3			
			LIG4	AtLIG4	AT5G57160	(Waterworth et al., 2010)
				LIG6	AT1G66730	(Waterworth et al., 2010)

## Base Removal

BER is initiated by DNA glycosylases that recognize and excise the modified or damaged bases by hydrolytic cleavage of the N-glycosidic bond between the C1′ of the 2′-deoxyribose and the N atom at the target base. Most DNA glycosylases studied to date remove the target base through a base-flipping mechanism that involves DNA bending and distortion to facilitate base extrusion. Then, the damaged base is inserted into a hydrophobic pocket so that catalytic residues can access the N-glycosidic bond, and an amino acid (the *base flipper* residue) fills in the vacant space left behind in the double helix. In some cases, the intercalated residue and/or other enzyme residues make specific interactions with the orphan opposite base in the complementary strand (Huffman et al., 2005; Dalhus et al., 2009). Monofunctional DNA glycosylases cleave the N-glycosidic bond using an activated water molecule as nucleophile to attack the C1′ of the target nucleotide, whereas bifunctional DNA glycosylases use as nucleophile the amine moiety of a residue from the active site, thereby forming a Schiff base intermediate.

There are different types of DNA glycosylases, each specialized for a particular type of chemical damage or a range of structurally related lesions. Five structural superfamilies of DNA glycosylases have been identified: uracil DNA glycosylase (UDG), alkyladenine DNA glycosylase (AAG), helix-hairpin–helix (HhH-GPD), helix–two-turn–helix (H2TH), and HEAT-like repeat (HLR) (Dalhus et al., 2009). Despite their different structures, it seems clear that all DNA glycosylase families, except the HLR family (Mullins et al., 2015), use a base-flipping strategy to recognize and excise their substrates. Since HLR-like DNA glycosylases are mostly prokaryotic and not present in plants, in the following sections, we will concentrate on the remaining four superfamilies.

### UDG Superfamily

Uracil DNA glycosylases (UDG) are monofunctional glycosylases that remove uracil from DNA. In addition to spontaneous deamination of cytosine to uracil, which contributes significantly to the accumulation of mutagenic U:G mispairs, dUMP can be misincorporated during replication in U:A pairs (Kavli et al., 2007). UDG activity has been partially purified in some plant species, such as carrot, wheat, onion, or maize (Blaisdell and Warner, 1983; Maldonado et al., 1985; Bensen and Warner, 1987; Talpaert-Borle, 1987; Bones, 1993).

All members of the UDG superfamily are proteins with a single domain comprising four-stranded parallel twisted β-sheet flanked by α-helices (Mol et al., 1995). On the basis of substrate specificity, UDGs are classified into six families distributed across eubacteria, archaea, yeast, animals, and plants (Schormann et al., 2014). Family 1 of UDG, represented by *Escherichia coli* Ung and human UNG, is the most extensively studied and the most widely distributed, present in most species examined, with some remarkable exceptions such as *Drosophila melanogaster* and Archaea (Aravind and Koonin, 2000).

A member of the Family-1 UDG from *Arabidopsis*, AtUNG (AT3G18630), has been purified and characterized (Cordoba-Cañero et al., 2010). The AtUNG protein sequence conserves the active site motifs A and B present in the five UDG families and the critical residues implicated in base recognition and catalysis in Family-1 enzymes (Cordoba-Cañero et al., 2010). In human cells, two isoforms of UNG, with different cellular localizations, are generated by alternative splicing: UNG1 in the mitochondria and UNG2 in the nucleus (Nilsen et al., 1997). The N-terminal sequence of AtUNG contains a putative PCNA-binding motif and shows higher degrees of similarity to human UNG2 than to UNG1 (Cordoba-Cañero et al., 2010). So far, no evidence of AtUNG multiple targeting has been found in *Arabidopsis*, although UDG activity has been detected in mitochondrial extracts and an AtUNG-eGFP fusion protein, transiently expressed in *N. benthamiana* leaves, colocalized with mitochondria in protoplasts generated from the agro-infiltrated tissues (Boesch et al., 2009). Therefore, the possibility that plant UNG is targeted to mitochondria and/or chloroplasts cannot be ruled out.

*E. coli* and human UNG excise uracil but no other 5-substituted pyrimidines, except for 5-fluorouracil (5-FU) (Mauro et al., 1993; Krokan et al., 2002), probably because uracil and 5-FU residues are small enough to fit the tight uracil-binding pocket compared to the larger chloro-, methyl-, bromo-, and iodo-substituted uracils (Liu et al., 2002). In contrast to bacterial and human enzymes, AtUNG lacks detectable activity on 5-FU (Cordoba-Cañero et al., 2010), suggesting that steric constraints imposing selectivity and specificity for uracil against other pyrimidines are more strict in the plant enzyme.

Available evidence suggests that AtUNG encodes the major UDG activity detected in *Arabidopsis* cell extracts, since such activity disappears in *atung* null mutants (Cordoba-Cañero et al., 2010). Similarly to other multicellular organisms, *atung* mutant plants show neither visible phenotypic alterations nor detectable increased levels of uracil in the genome, although neither UDG activity nor uracil BER is detected (Cordoba-Cañero et al., 2010). However, inactivation of the *AtUNG* gene protects plants against the cytotoxic effect of 5-FU, indicating that UDG activity is harmful for cells with high levels of dUTP/dTTP ratio (Cordoba-Cañero et al., 2010). The *Arabidopsis* genome contains another gene (AT2G10550) with partial sequence similarity to UNG, and it has been suggested that it is an inactive paralog interrupted by two transposon insertions, probably originated by a gene duplication process (Cordoba-Cañero et al., 2010). UDG Family 2 (exemplified by *E. coli* Mug and human TDG), Family 3 (typified by vertebrate SMUG1), and Families 4 and 5 (identified in thermophilic bacteria and archaea) are not represented in plants (Cordoba-Cañero et al., 2010).

### AAG Superfamily

The members of the AAG superfamily, also known as alkylpurine-DNA glycosylases or N-methylpurine DNA glycosylases, are compact single-domain enzymes with a mixed α/β structure and a positively charged DNA-binding surface (Brooks et al., 2013). These enzymes, unrelated to other BER enzymes, are monofunctional glycosylases that remove alkylated purines and ethenopurines, and the best characterized is human AAG (hAAG). In land plants, a hAAG ortholog (AtAAG) was first isolated in *Arabidopsis* (Santerre and Britt, 1994). AtAGG complements the sensitive phenotype to methyl methanesulfonate (MMS) of an *E. coli* double mutant deficient in N3-methyladenine (N3-meA) glycosylases and excises N3-meA, but not N7-methylguanine (N7-meG) (Santerre and Britt, 1994; Malhotra and Sowdhamini, 2013). Expression of *AtAAG* seems to be higher in growing tissues, supporting the importance of maintaining genome integrity in dividing cells (Santerre and Britt, 1994; Shi et al., 1997). AAG genes have been also detected in other higher plants, including maize (Fu et al., 2010; Wang et al., 2015), wheat (Mak et al., 2006), grape (Tillett et al., 2012), and *Brachypodium distachyon* (Kim et al., 2012).

### HhH-GPD Superfamily

The HhH-GPD superfamily is the most heterogenous DNA glycosylase superfamily, with widely different substrate specificities. Its characteristic HhH motif is a DNA-binding domain that is present in a number of proteins that bind DNA in a sequence-independent manner (Thayer et al., 1995; Doherty et al., 1996). This superfamily includes both monofunctional and bifunctional members, and their structures share two characteristic domains with the active site located at their junction. The core fold consists of four N-terminal and six to seven C-terminal α-helices, linked by a type-II β-hairpin (Doherty et al., 1996). The HhH motif is followed by a loop (GPD motif) containing glycine (G), proline (P), and an invariable aspartic acid (D) residue (Huffman et al., 2005). The conserved aspartic acid activates the nucleophile (a molecule of water or a lysine residue in monofunctional or bifunctional DNA glycosylases, respectively) for attack of the N-glycosidic bond (Huffman et al., 2005). These enzymes remove a broad spectrum of lesions, including those generated by alkylation, oxidation, or hydrolytic damage.

Mammals do not appear to possess homologs of the 3-methyladenine DNA glycosylases belonging to this family (Tag and AlkA), and rather, they use AAG to remove alkylated purines (Dalhus et al., 2009). However, in addition to AtAAG *Arabidopsis* possesses 9 and 2 putative homologs of Tag and AlkA enzymes, respectively (Britt, 2002), none of which has been characterized so far.

Oxidatively damaged pyrimidines in *E. coli* are repaired by Nth, also known as Endonuclease III (EndoIII), a bifunctional glycosylase with AP lyase activity (Katcher and Wallace, 1983). *Arabidopsis* possesses two structural and functional homologs of Nth: AtNTH1 (AT2G31450) (Roldan-Arjona et al., 2000) and AtNTH2 (AT1G05900) (Gutman and Niyogi, 2009). AtNTH1 exhibits DNA glycosylase activity on urea and thymine glycol from double-stranded DNA and also possesses AP lyase activity (Roldan-Arjona et al., 2000). AtNTH2 has three splice variants described. Expressed AT1G05900.2 splice variant exhibited significant glycosylase/lyase activity on DNA containing thymine glycol (Gutman and Niyogi, 2009). AtNTH1 and AtNTH2 (AT1G05900.2 splice variant) fused to GFP seem to be targeted to chloroplast nucleoids (Gutman and Niyogi, 2009). An alternative AtNTH1 transcription initiation site would allow translation from a downstream ATG to generate a predicted protein with a putative nuclear localization signal and lacking chloroplast targeting (Roldan-Arjona et al., 2000; Gutman and Niyogi, 2009). A phylogenetic analysis of EndoIII homologs in bacteria, archaea, and eukaryotes reveals major phylogenetic relationships of AtNTH1 with eukaryotic proteins, being most similar to EndoIII from *Schizosaccharomyces pombe* (Roldan-Arjona et al., 2000). In *Saccharomyces cerevisiae*, there are also two functional homologs (Ntg1p and Ntg2p) of *E. coli* EndoIII, with Ntg1p localizing primarily to mitochondria and Ntg2p to the nucleus (You et al., 1999). In humans, however, the only functional homolog identified so far (hNTH1) contains a putative nuclear localization signal at the N-terminus (Aspinwall et al., 1997), although it has been located in both nucleus and mitochondria (Takao et al., 1998). The subcellular localization of other splice variants of AtNTH2 remains to be determined. Therefore, AtNTH1 and AtNTH2 could have a role in the removal of oxidative lesions in both nuclear and organellar genomes.

The major oxidation product of purines is 7-hydro-8-oxoguanine (8-oxoG), which is originated as a consequence of the oxidation of the hydroxyl radical of C8 of a guanine (Dizdaroglu, 1985). It is a highly mutagenic lesion due to its capacity to pair with both cytosine and adenine (Shibutani et al., 1991). Repair of 8-oxoG in eukaryotes is performed by 8-oxoguanine DNA glycosylases (OGG), bifunctional glycosylases belonging to the HhH-GPD superfamily, that catalyze the excision of 8-oxoG and cleave the generated AP site by a β-elimination mechanism (Girard and Boiteux, 1997). Ogg1 homologs are present in eukaryotes, including humans (Radicella et al., 1997; Roldan-Arjona et al., 1997), and in some archaea, but not in bacteria (Eisen and Hanawalt, 1999). *Arabidopsis* has an OGG1 homolog with more than 40% identity with yeast and human OGG1 proteins (Dany and Tissier, 2001; Garcia-Ortiz et al., 2001). In contrast with the mammalian *OGG1* gene that produces several splice variants with mitochondrial or nuclear localization (Nishioka et al., 1999), in *Arabidopsis*, only one isoform of this protein seems to be produced (Dany and Tissier, 2001). The *Arabidopsis* OGG1-predicted protein possesses a putative nuclear localization signal at the N-terminus, but lacks identifiable signal sequences for targeting to plastids or mitochondria (Dany and Tissier, 2001; Garcia-Ortiz et al., 2001). Although it has been suggested that there is a putative mitochondrial targeting sequence in MtOGG1 from *Medicago truncatula* (Macovei et al., 2011), the subcellular localization of OGG1 in plants remains to be determined.

Expression of AtOGG1 abolishes the mutator phenotype of an *E. coli mutM mutY* mutant strain, thus indicating its capacity to excise 8-oxoG *in vivo* (Dany and Tissier, 2001; Garcia-Ortiz et al., 2001). *Arabidopsis atogg1* mutants show no obvious phenotypic differences in comparison with wild-type plants (Murphy, 2005), but *in vitro* BER assays with *atogg1* mutant cell extracts show that AtOGG1 contributes to the excision of 8-oxoG and counteracts accumulation of oxidative DNA damage (Cordoba-Cañero et al., 2014). Biochemical characterization of AtOGG1 demonstrated its activity on DNA substrates containing 8-oxoG (Dany and Tissier, 2001; Garcia-Ortiz et al., 2001) and the imidazole ring-opened derivative 2,6-diamino-4-hydroxy-5-formamidopyrimidine (FapyGua) (Morales-Ruiz et al., 2003). The enzyme preferentially excises 8-oxoG paired to guanine, in comparison with 8-oxoG:A pairs generated with high frequency during replication (Morales-Ruiz et al., 2003). In *E. coli*, the excision of A mispaired to 8-oxoG is catalyzed by MutY (Michaels et al., 1992). Homologs to bacterial *mutY* have been characterized in both eukaryotes and archaea (Eisen and Hanawalt, 1999). *Arabidopsis* possesses a putative MutY homolog (AT4G12740), which remains uncharacterized.

Spontaneous deamination of 5-methylcytosine (5-meC) to thymine leads to T:G mispairs targeted by thymine-DNA mismatch glycosylases, such as bacterial MIG and mammalian MBD4 (also known as MED1) (Horst and Fritz, 1996; Hendrich et al., 1999; Berti and McCann, 2006). MBD4, which possesses a methyl-CpG-binding domain (MBD) and a HhH-GPD DNA glycosylase domain, is a monofunctional DNA glycosylase that excises U or T mispaired to G, with a preference for mismatches at a CpG context (Nash et al., 1996; Hendrich and Bird, 1998; Bellacosa et al., 1999; Hendrich et al., 1999; Petronzelli et al., 2000a; Petronzelli et al., 2000b; Turner et al., 2006). A plant MBD4 homolog, termed MBD4-like (AtMBD4L, AT3G07930), has been identified in *Arabidopsis* (Ramiro-Merina et al., 2013). AtMBDL4 and other plant MBD4 homologs lack the MBD domain present at the N-terminus of metazoan MBD4 proteins, but share a C-terminal catalytic domain with critical residues specifically conserved in MBD4 glycosylases. AtMBD4L excises uracil and 5-substituted uracil derivatives, such as 5-BrU or 5-FU, with more efficiency than thymine (Ramiro-Merina et al., 2013). Since AtMBD4L shows a clear preference for a CpG sequence context, where the majority of plant DNA methylation takes place, it has been suggested that this enzyme plays a role in preventing the potential mutagenic effects of 5-meC deamination (Ramiro-Merina et al., 2013). Four alternative splice variants of AtMBD4L have been described, two of which (AtMBD4L3 and AtMBD4L4) are expressed in leaves and flowers, whereas another one (AtMBD4L3) has been localized in the nucleus (Nota et al., 2015). Interestingly, plants overexpressing AtMBD4L3 show increased expression of AtLIG1 (Nota et al., 2015).

#### DML Family

The DEMETER-LIKE (DML) family is a plant-specific DNA glycosylase family belonging to the HhH-GPD superfamily. Its founding members are four *Arabidopsis* proteins: DME (DEMETER), ROS1 (REPRESSOR OF SILENCING 1), DME-like 2 (DML2), and DME-like 3 (DML3) (Choi et al., 2002; Gong et al., 2002; Ortega-Galisteo et al., 2008). All four enzymes are 5-meC DNA glycosylases/lyases involved in active DNA demethylation through a BER process. Proteins from the DML family appear to be unique to plants, with putative orthologs present in mosses (*Phycomitrella patens*) and unicellular green algae (*Ostreococcus*, for example), suggesting that active demethylation through excision of 5-meC may have appeared early during plant evolution (Roldan-Arjona and Ariza, 2009a).

All DML proteins possess an HhH-GPD motif with the invariant aspartate, a conserved lysine residue characteristic of bifunctional DNA glycosylases, and a [4Fe–4S] cluster. They are very large proteins, ranging from 1,100 to 2,000 amino acids, in comparison to other members of the HhH-GPD superfamily (200–400 amino acids). One of its distinctive characteristics is their discontinuous catalytic domain, comprised of two conserved regions separated by a predicted unstructured sequence whose length varies across family members (Ponferrada-Marin et al., 2011). They also contain a conserved carboxy-terminal domain, that is not related with any known protein family (Choi et al., 2002; Gong et al., 2002; Morales-Ruiz et al., 2006) but is required for catalytic activity (Ponferrada-Marin et al., 2010; Hong et al., 2014), and a short amino-terminal domain significantly rich in lysine that facilitates demethylation in long substrates (Ponferrada-Marin et al., 2010). In addition to 5-meC, ROS1, DME, and DML3 excise T mispaired with G and show a preference for CpG contexts (Morales-Ruiz et al., 2006; Ortega-Galisteo et al., 2008), thus supporting an additional DNA repair role similar to that of MBD4L in counteracting the mutagenic consequences of 5-meC deamination.

Members of the DML family are bifunctional DNA glycosylase/lyases that excise the target base and cleave the phosphodiester backbone by β- or β, δ-elimination, generating a single-nucleotide gap with the 3′-PUA or 3′-P termini, respectively (Agius et al., 2006; Gehring et al., 2006; Morales-Ruiz et al., 2006; Penterman et al., 2007; Ortega-Galisteo et al., 2008). Such 3′-blocked ends must be processed to the 3′-OH termini before a DNA polymerase and a DNA ligase may fill and seal the gap, respectively.

### H2TH Superfamily

Proteins of the H2TH superfamily (also known as Fpg/Nei) are characterized by a common structure comprising of domains separated by a flexible linker sequence. The catalytic amino acid that acts as nucleophile is a conserved proline located at the N-terminal domain, whereas the C-terminal domain contains a zinc finger required for DNA binding (Sugahara et al., 2000). All of them are bifunctional DNA glycosylases that cleave the sugar–phosphate backbone by β, δ-elimination activity, and they are mostly involved in the repair of oxidative damage (Fromme and Verdine, 2004; Huffman et al., 2005). The two founding members of the H2TH superfamily are the *E. coli* proteins Formamidopyrimidine DNA glycosylase (Fpg, also known as MutM) and Endonuclease VIII (Nei). Fpg recognizes formamidopyrimidines, 8-oxoG, as well as its oxidation products guanidinohydantoin (Gh), and spiroiminodihydantoin (Sp), whereas Nei primarily acts on damaged pyrimidines (Kathe et al., 2009).

Phylogenetic analysis has confirmed that both Fpg and Nei homologs are widely distributed in prokaryotes. In eukaryotes, Fpg homologs are only found in plant and fungi clades, whereas Nei homologs are restricted to metazoans, although they have been lost in many non-vertebrate lineages (Kathe et al., 2009). Mammals possess three Nei-like proteins (NEIL1, NEIL2, and NEIL3) (Wallace, 2013).

Although plants have both Ogg and Fpg homologs (Ohtsubo et al., 1998; Dany and Tissier, 2001; Garcia-Ortiz et al., 2001; Scortecci et al., 2007; Macovei et al., 2011), the relative roles of these two types of enzymes in counteracting oxidative DNA damage are not well understood. Alternative splicing of *Arabidopsis* FPG leads to seven different isoforms, and two of them show variation in the expression levels depending on the analyzed tissue (Ohtsubo et al., 1998; Murphy and Gao, 2001). AtFPG1 is the only isoform characterized biochemically, and whereas its activity excising 8-oxoG was almost undetectable, it shows a potent AP lyase activity (Kathe et al., 2009). The inability of AtFPG1 to excise 8-oxoG has been attributed to the presence of a very short version of the a-F-b9/10 loop, which is involved in 8-oxoG recognition (Duclos et al., 2012).

T-DNA insertion mutant plants lacking both AtFPG and AtOGG proteins do not show any obvious phenotype distinguishable from the wild type (Murphy, 2005). However, there is evidence that both enzymes participate in 8-oxoG repair and contribute to counteract the oxidative DNA damage in *Arabidopsis* (Cordoba-Cañero et al., 2014). Interestingly, *atfpg atogg1* double mutants show increased levels of oxidative DNA damage not only in the nucleus but also in the mitochondria (Cordoba-Cañero et al., 2014).

## AP Site Incision

AP sites are frequently found in DNA due to the spontaneous hydrolysis of the N-glycosylic bond. Additionally, they are also repair intermediates generated by monofunctional DNA glycosylases during BER (**Figure 1**). It has been estimated that more than 10,000 bases are lost spontaneously per day per mammalian cell, being purines much more susceptible to spontaneous loss than pyrimidines (Lindahl and Nyberg, 1972). AP sites are DNA lesions with cytotoxic effects due to their capacity to block DNA replication and transcription, but also have potential mutagenic consequences if they are bypassed by DNA polymerases (Loeb, 1985; Prakash et al., 2005). AP site repair is initiated by either AP endonucleases or AP lyases, generating single-strand breaks (SSB) with either 5′- or 3′-blocked ends, respectively, that cannot be used as substrates by DNA polymerases or DNA ligases. Such SSBs can be converted into highly toxic double-strand breaks (DSB) if not processed before DNA replication (Caldecott, 2001).

### AP Endonucleases

AP endonucleases recognize AP sites and perform hydrolysis at their 5′-side, yielding SSBs with 3′-OH and 5′-dRP ends (Levin and Demple, 1990) (**Figure 1**). Based on structural folding and amino acid sequence similarity to the major AP endonucleases of *E. coli*, these enzymes are classified under Endonuclease IV (EndoIV, also known as Nfo) and Exonuclease III (ExoIII) families. Under physiological conditions, ExoIII is responsible for the vast majority of AP endonuclease activity detected in *E. coli* (Weiss, 1976), whereas EndoIV is induced during oxidative stress (Chan and Weiss, 1987). Although EndoIV and ExoIII families have overlapping DNA substrate specificities, they are distinguished by their modes of DNA damage recognition (Redrejo-Rodriguez et al., 2016). Moreover, their tertiary structure and their divalent metal requirements are completely different; while ExoIII family proteins are Mg^2+^-dependent, EndoIV family members are Zn^2+^-dependent, indicating that they have evolved independently from different ancestors. Importantly, ExoIII family members are present in all kingdoms of life, while EndoIV members are absent in some groups, such as mammals and plants (Daley et al., 2010). An EndoIV homolog in *S. cerevisiae* (Apn1) has been identified as the main AP endonuclease activity in this species (Popoff et al., 1990). In *S. pombe*, an EndoIV homolog exists, too, but seems to play only a backup role in DNA repair (Ramotar et al., 1998).

Mammalian genomes encode two proteins, APE1 and APE2 (also known as APEX1 and APEX2), with sequence similarity to ExoIII. APE1 is the major AP endonuclease activity, performing more than 95% of total AP site incision (Demple and Sung, 2005), whereas the activity of APE2 is significantly lower (Hadi and Wilson, 2000). APE1 possesses a C-terminal region responsible for interaction with DNA and AP endonuclease activity (Fritz, 2000) and a unique N-terminal region, absent in ExoIII, required for a redox activity regulating the DNA-binding potential of several transcription factors (Georgiadis et al., 2008).

The *Arabidopsis* genome encodes three AP endonuclease homologs of ExoIII: APE1L, ARP, and AtAPE2. APE1L (AT3G48425) and ARP (AT2G41460) are similar to the major human AP endonuclease APE1, and AtAPE2 (AT4G36050) is similar to the human APE2 (Murphy et al., 2009). Homologous sequences have been identified also in sugarcane (Maira et al., 2014; Cabral Medeiros et al., 2019) and rice (Joldybayeva et al., 2014).

Like its human APE1 homolog, *Arabidopsis* ARP possesses a repair-independent redox activity able to regulate the DNA-binding capacity of some transcription factors (Babiychuk et al., 1994). On the other hand, its DNA incision activity is essential during uracil or AP site repair *in vitro* (Cordoba-Cañero et al., 2011). ARP also processes AP sites generated by AtFPG and/or AtOGG1 during 8-oxoG repair and performs an important role in repairing oxidative DNA damage accumulated during seed aging (Cordoba-Cañero et al., 2014). Several T-DNA insertion mutants in *ARP* show no phenotypic differences with wild-type plants (Gutman and Niyogi, 2009; Murphy et al., 2009; Cordoba-Cañero et al., 2011), despite the fact that ARP acts as a protective factor when levels of uracil in DNA are artificially increased by 5-FU treatment (Cordoba-Cañero et al., 2011). ARP fusion proteins to GFP are targeted to chloroplasts, and the capacity of chloroplast protein extracts to incise osmium tetroxide-treated DNA is reduced in *Arabidopsis arp* mutants (Gutman and Niyogi, 2009).

All three AP endonucleases from *Arabidopsis* have been biochemically characterized by several groups (Lee et al., 2014; Li et al., 2015; Li et al., 2018). AP endonuclease activity of ARP, APE1L, and AtAPE2 has been demonstrated, with AtAPE2 activity the weakest (Lee et al., 2014; Li et al., 2015). Unlike human APE1, ARP discriminates between AP sites generated by spontaneous base loss or by enzymatic excision. Thus, ARP cleaves AP sites generated by N7-meG excision but is unable to process AP sites originated due to spontaneous depurination of N7-meG, suggesting that these two types of AP sites possess different chemical or structural properties not yet identified (Barbado et al., 2018). In addition to AP endonuclease activity, AP endonucleases are endowed with phosphodiesterase and/or phosphatase activities involved in cleaning blocked DNA ends (see the section *Cleaning of DNA Termini*).

Whereas deletion of the *APE1* gene results in very early embryonic lethality in mice (Xanthoudakis et al., 1996), *Arabidopsis* T-DNA insertional mutants of APE1L, AtAPE2, or ARP display no phenotypic defects (Murphy et al., 2009). However, the simultaneous inactivation of APE1L and AtAPE2 leads to a seed abortion phenotype, whereas a joint deficiency with either APE1L or AtAPE2 does not cause any effect. These results indicate that APE1L and AtAPE2 are probably performing overlapping functions required for seed viability (Murphy et al., 2009), likely in repair of DNA damage generated during seed development and/or the 3′-blocked ends generated by DML DNA glycosylases during active DNA demethylation (see the section *DML Family*). Although ARP is dispensable for normal seed development, it performs a protective role against the adverse effects of seed aging (Cordoba-Cañero et al., 2014).

### AP Lyases

Although it has been widely assumed that AP sites are mainly processed by AP endonucleases, accumulating evidence points to an additional important role for AP lyases. For example, in both *S. cerevisiae* and *S. pombe*, AP sites are first incised by the AP lyase activity of Nth1 homologs, which produce 3′-PUA blocked termini that are subsequently processed by AP endonucleases (Pascucci et al., 2002; Li et al., 2015). Evidence of an important role of AP lyases in the processing of abasic sites has also been reported recently in plants. In *Arabidopsis*, spontaneous depurination of MMS-induced N7-meG generates AP sites that are not recognized by ARP (see above) and are exclusively repaired through an AP endonuclease-independent route initiated by the AP lyase activity of AtFPG (Barbado et al., 2018). AtFPG is the major, possibly the only, AP lyase activity detectable in *Arabidopsis* cell extracts (Barbado et al., 2018). AP site incision catalyzed by AtFPG generates a 3′-P end that is converted to 3′-OH by the DNA 3′-phosphatase ZDP (see the section *Blocked 3′-Termini*) before repair is completed (Barbado et al., 2018).

## Cleaning of DNA Termini

### Blocked 3′-Termini

Blocked 3′-termini arise from the incision activity of bifunctional DNA glycosylases/AP lyases. Incisions performed by β-elimination generate 3′-PUA blocked ends, whereas those caused by β, δ–elimination produce 3′-P ends (**Figure 1**).

Human APE1 possesses 3′-phosphodiesterase activity to remove 3′-PUA blocked ends and also exhibits a weak 3′-phosphatase activity (Demple and Harrison, 1994; Suh et al., 1997). In contrast, human APE2 has weak AP endonuclease activity but potent 3′-phosphodiesterase and 3′→5′-exonuclease activities (Burkovics et al., 2006).

In *Arabidopsis*, APE1L is able to efficiently process the 3′-PUA ends *in vitro* (Lee et al., 2014; Li et al., 2015). Furthermore, APE1L has been demonstrated to function in the active DNA demethylation pathway by processing the 3′-PUA termini generated by the bifunctional 5-meC DNA glycosylases/lyases of the DML family (Li et al., 2015). It has been also shown that APE1L and APE2 possesses 3′-phosphatase activity *in vitro* (Li et al., 2015; Li et al., 2018). The wheat homolog of APE1L possesses a weak AP endonuclease activity, as compared to human APE1, but displays 3′-phosphodiesterase, 3′-phosphatase, and 3′→5′ exonuclease activities (Joldybayeva et al., 2014). It has been also demonstrated that *Arabidopsis* ARP exhibits NIR (Nucleotide Incision Repair) and 3′→5′ exonuclease activities (Akishev et al., 2016).

When BER is initiated by bifunctional DNA glycosylases that perform β, δ-elimination, a gap flanked by phosphates is generated (**Figure 1**). The 3′-P blocked end is not a substrate for DNA polymerases, and AP endonucleases seem not to be efficient 3′-phosphatases. In mammalian BER, this problem is solved using polynucleotide kinase/3′-phosphatase (PNKP) for 3′-P removal (Jilani et al., 1999). Mammalian PNK functions in AP endonuclease-independent BER of oxidative DNA damage (Wiederhold et al., 2004) as well as in SSBs and DSBs repair (Whitehouse et al., 2001; Chappell et al., 2002).

In plants, proteins with 3′-DNA phosphatase activity have been described in maize (ZmDP2) and *Arabidopsis* (ZDP, zinc finger DNA 3′-phosphoesterase, AT3G14890). They show partial sequence similarity to mammalian PNKP, but lack the associated 5′-kinase activity, suggesting that, unlike PNKP, they are unable to phosphorylate the 5′-hydroxyl termini at SSBs (Betti et al., 2001; Petrucco et al., 2002; Martinez-Macias et al., 2012).

ZDP, which apparently is the only enzyme responsible for the DNA 3′-phosphatase activity detectable in *Arabidopsis* cell extracts, participates in the processing of the 3′-P ends generated by AtFPG and AtOGG1 during 8-oxoG repair, as well as those produced by the 5-meC DNA glycosylases ROS1 and DME during the active DNA demethylation BER pathway (Martinez-Macias et al., 2012; Cordoba-Cañero et al., 2014). Mutants deficient in ZDP do not display any phenotypic alteration under normal growth conditions, but show hypersensitivity to MMS (Martinez-Macias et al., 2012). As indicated above, AP sites generated by nonenzymatic release of MMS-induced N7-meG are cleaved by AtFPG, and the generated 3′-P is processed by ZDP. In fact, *zdp*-deficient plants possessing an additional *fpg* mutation partially recover MMS resistance, suggesting that unrepaired AP sites are less toxic than downstream SSB repair intermediates with blocked 3′-P ends (Barbado et al., 2018).

### Blocked 5′-Termini

When abasic sites are incised by AP endonucleases, a gap flanked by a 3′-OH group and a 5′-dRP blocked terminus is generated (**Figure 1**). To continue the repair pathway, the 5′-dRP end is processed to a 5′-P end by a dRP lyase activity. In mammals, the major dRP lyase activity is associated to DNA Polymerase β (Srivastava et al., 1998), through an N-terminal 8-kDa domain characteristic of Family X of DNA polymerases (Beard and Wilson, 2000). Processing of 5′-dRP may be rate limiting, and this blocking group may be also removed by strand displacement and incision during the LP-BER sub-pathway (**Figure 1**) (see the section *Gap Filling: Short-Patch and Long-Patch BER Sub-pathways*).

Unlike mammals, plants and yeast do not possess DNA polymerase β orthologs, but have related enzymes termed Pol λ and Pol IV, respectively. Pol λ, which is also present in mammalian cells, belongs to the X-family of DNA polymerases, shares more than 30% of sequence homology with mammalian Pol β (Garcia-Diaz et al., 2000) and also displays DNA polymerase and dRP lyase activities (Garcia-Diaz et al., 2000; Garcia-Diaz et al., 2002). Like Pol IV in yeast, Pol λ is the only member of the Family X of DNA Polymerases present in most plants. However, sequences with similarity to X-family members Pol μ and TdT have been identified in the unicellular alga *Chlamydomonas reinhardtii* (Morales-Ruiz et al., 2018). It has been shown that human Pol λ possesses dRP lyase activity (Garcia-Diaz et al., 2001), and it can function as a backup enzyme for DNA Pol β in BER (Braithwaite et al., 2010). The role of plant Pol λ has been studied in rice and *Arabidopsis* (Uchiyama et al., 2004; Amoroso et al., 2011; Roy et al., 2011). The rice Pol λ ortholog has been partially characterized, and biochemical analysis indicates that it possesses dRP lyase activity (Uchiyama et al., 2004). Although some biochemical properties of *Arabidopsis* Pol λ have been described, there is no evidence reported of its dRP lyase activity (Amoroso et al., 2011; Roy et al., 2011).

In addition to Pol β and Pol λ, Pol θ, other human DNA polymerase that belongs to Family A, possesses dRP lyase activity, and it has been demonstrated to function in human BER (Prasad et al., 2009). It has been suggested that although human Pol θ is not essential in BER, it may be a backup enzyme, and the same may be true in plants. In *Arabidopsis*, the gene *TEBICHI* (*TEB*) codes for a Pol θ homolog. Inactivation of *TEB* causes sensitivity to DNA-damaging agents, such as mitomycin C and MMS, that promote DNA crosslinks and SSBs/DSBs, respectively (Inagaki et al., 2006; Inagaki et al., 2009). Nevertheless, there is no data available supporting an implication of AtPolθ in dRP processing during BER in plants.

The *Arabidopsis* genome encodes two family-A DNA Polymerase paralogs, AtPolIA and AtPolIB, which are the only DNA Polymerases in plant organelles identified to date. Both have been implicated in organellar DNA replication, whereas only AtPolIB, but not AtPolIA, is involved in organellar DNA repair (Ono et al., 2007; Parent et al., 2011). Recently, the capacity of both AtPolIA and AtPolIB to remove the 5′-dRP moiety by an intrinsic lyase activity it has been described (Trasvina-Arenas et al., 2018).

## Gap Filling: Short-Patch and Long-Patch BER Sub-Pathways

Gap filling during BER may proceed either *via* short-patch (SP), by incorporation of only a single nucleotide, or long-patch (LP), by insertion of 2 to 13 nucleotides (**Figure 1**). In mammalian cells the contribution of DNA Pol β and DNA Ligase III in SP-BER has been demonstrated (Kubota et al., 1996), and since plants lack homologs of both enzymes, it was initially accepted that plants only perform LP-BER (Uchiyama et al., 2008). Nevertheless, it has been confirmed that *Arabidopsis* cell extracts repair uracil and AP sites by both SP- and LP-DNA synthesis (Cordoba-Cañero et al., 2009; Cordoba-Cañero et al., 2011). As indicated above, Pol λ is the only member of Family X of DNA polymerases in plants. Although functions of plant Pol λ in nucleotide excision repair (Roy et al., 2011), oxidative DNA damage bypass (Amoroso et al., 2011), non-homologous end joining (Roy et al., 2013; Furukawa et al., 2015), and DSB repair (Sihi et al., 2015) have been stablished, its role, if any, in SP-BER remains to be clarified.

The alternative BER sub-pathway, LP-BER, occurs when two or more nucleotides are inserted in the repair gap. In mammals, Pol β is able to incorporate the first nucleotide in LP-BER (Podlutsky et al., 2001), but the elongation step is performed by replicative DNA Polymerases, such as DNA Pol δ and Pol ε. Plants possess orthologs of both DNA polymerases δ and ε, and evidences obtained in rice and *Arabidopsis* demonstrate the important role of Pol ε in DNA replication (Uchiyama et al., 2002; Ronceret et al., 2005). However, their involvement in LP-BER remains to be determined.

It has been suggested that the choice between SP- and LP-BER could be influenced by the nature of the lesion and/or the DNA glycosylase that initiates BER, and that the equilibrium between both sub-pathways may be additionally affected by the phase of the cell cycle (Fortini and Dogliotti, 2007). In *Arabidopsis*, the choice between SP- and LP-BER is affected by the nature of the 5′-end of the repair gap. When the 5′-end is a reduced dRP not amenable to β-elimination by dRP lyases, the SP-BER sub-pathway is abrogated, and repair is performed exclusively by LP-BER (Cordoba-Cañero et al., 2009; Cordoba-Cañero et al., 2011). Also, it has been demonstrated in *Arabidopsis* that AP sites generated by spontaneous depurination of N7-meG are repaired by SP-BER, whereas those generated enzymatically can be repaired by both SP- and LP-BER (Barbado et al., 2018).

DNA polymerases performing LP-BER promote strand displacement and generate a 5′-end single-stranded “flap” that needs to be removed by endonucleolytic cleavage. In mammals, this step is performed by Flap Endonuclease 1 (FEN1) (Kim et al., 1998), a structure-specific 5′ endo/exonuclease (Harrington and Lieber, 1994) belonging to the Rad2 nuclease family with essential roles in the processing of Okazaki fragments during replication and in LP-BER (Liu et al., 2004).

Plant homologs of FEN1 were first partially characterized in cauliflower (*Brassica oleracea* var. *botrytis*) inflorescences (Kimura et al., 1997) and later in rice [OsFEN1a and OsFEN1b (Kimura et al., 2000; Kimura et al., 2003)] and *Arabidopsis* [AtFEN1 (AT5G26680) (Zhang et al., 2016a; Zhang et al., 2016b)]. OsFEN1a and OsFEN1b proteins show a high degree of sequence similarity, and analysis of their expression revealed correlation with cell proliferation (Kimura et al., 2003). However, only OsFEN1a is able to complement *S. cerevisiae* null mutants deficient in the FEN1 homolog *rad27* (Reagan et al., 1995; Kimura et al., 2003). Similarly, *Arabidopsis* AtFEN1 partially complements a *rad27* mutant. OsFEN-1a possesses both 5′-endonuclease and 5′-exonuclease activities (Kimura et al., 2000), but AtFEN1 lacks exonuclease activity (Zhang et al., 2016a; Zhang et al., 2016b). Rice and *Arabidopsis* FEN1 homologs have been localized to the nucleus, and interaction between OsFEN-1a and PCNA has been reported (Kimura et al., 2001; Zhang et al., 2016a).

Whereas the knockout mutant of FEN1 causes early embryonic lethality in mice (Kucherlapati et al., 2002), yeast mutants are viable and show increased sensitivity to UV light and mutagens (Reagan et al., 1995; Vallen and Cross, 1995). In plants, AtFEN1 seems to be essential since no homozygous *Arabidopsis* mutants could be obtained from the progeny of a heterozygous *fen1-2* T-DNA insertion mutant (Zhang et al., 2016a). Shade avoidance mutant 6 (*sav6*) plants, which contain a single point mutation that affect mRNA splicing efficiency of AtFEN1, are hypersensitive to ultraviolet (UV)-C radiation and DSB-inducing agents (Zhang et al., 2016b). Furthermore, another AtFEN1 mutant, with a single nucleotide substitution (*fen1-1*), shows hypersensitivity to MMS and exhibits shortened telomeres (Zhang et al., 2016a). However, no evidence has been yet reported for a role of plant FEN1 homologs in BER.

## Nick Ligation

The SP and LP-BER sub-pathways converge by generating the same product: a nick flanked by 3′-OH and 5′-P termini. The culminating BER step is the action of a DNA ligase that seals the nick by catalyzing formation of a phosphodiester bond. DNA ligases are grouped into two families, ATP- and NAD^+^-dependent ligases, according to whether catalysis is coupled with pyrophosphate hydrolysis of ATP or NAD cofactors. The NAD^+^-dependent DNA ligases are highly conserved enzymes identified only in eubacteria, whereas most eukaryotic DNA ligases, together with archaeal and bacteriophage enzymes, are ATP-dependent DNA ligases (Ellenberger and Tomkinson, 2008).

In *E. coli*, the NAD^+^-dependent DNA LigA functions in both DNA replication and BER. Eukaryotes generally possess three ATP-dependent DNA ligases (Lig I, Lig III, and Lig IV in mammals). Lig IV is implicated in non-homologous end joining (Baumann and West, 1998) and seems to have no role in BER. The final ligation step during mammalian LP-BER is performed by Lig I, which is also essential in DNA replication, and the complex formed by Lig III and the X-ray repair cross-complementing 1 (XRCC1) protein participates in SP-BER (Cappelli et al., 1997; Timson et al., 2000; Sleeth et al., 2004).

*Arabidopsis* also possesses three ligases, AtLIG1, AtLIG4, and AtLIG6, but lack a Lig III homolog. AtLIG1 and AtLIG4 are orthologs of mammalian Lig I and Lig IV, respectively, whereas AtLIG6 is a plant-specific DNA ligase (Bonatto et al., 2005). AtLIG4 has been implicated in double-strand break repair (West et al., 2000; van Attikum et al., 2003) and, together with AtLIG6, seems to be critical for seed viability (Waterworth et al., 2010). *Arabidopsis* mutants in *AtLIG1* are lethal, and plants with a diminished expression display important phenotypic defects and deficiencies in the repair of single- and double-strand DNA breaks (Waterworth et al., 2009). Moreover, it has been demonstrated that AtLIG1 is essential for both SP- and LP-BER in *Arabidopsis* cell extracts (Cordoba-Cañero et al., 2011).

The mammalian *LIG3* gene, unlike the *LIG1* and *LIG4* genes, encodes different DNA ligase polypeptides by alternative translation initiation with different cellular functions and, notably, encodes the only mitochondrial DNA ligase (Tomkinson and Sallmyr, 2013). In contrast, in yeast and plants, different translation initiation sites generate distinct isoforms of DNA ligase 1 found in the nuclei and mitochondria (Donahue et al., 2001; Sunderland et al., 2006). No AtLIG1 targeting to chloroplasts has been detected in *Arabidopsis*.

## Additional Proteins Involved in BER

In addition to the BER factors discussed above, there are additional proteins (**Table 2**) that increase BER efficiency and/or function in the coordination of the various BER stages.

**Table 2:** Additional proteins involved in base excision repair in yeast, humans, and *Arabidopsis*.

Function	*S. cerevisiae*	*H. sapiens*	*Arabidopsis*		
			Name	Gene ID	Reference
**Processivity factor**	Pol30p	PCNA	AtPCNA1	AT1G07370	
			AtPCNA2	AT2G29570	(Amoroso et al., 2011)
**Scaffolding**		XRCC1	AtXRCC1	AT1G80420	(Martinez-Macias et al., 2013)
**Nick sensing**		PARP1	AtPARP1	AT2G31320	(Boltz et al., 2014)
		PARP2	AtPARP2	AT4G02390	(Song et al., 2015)
		PARP3	AtPARP3	AT5G22470	(Rissel et al., 2014)

### Proliferating Cell Nuclear Antigen (PCNA)

PCNA is an accessory factor that endows eukaryotic replicative polymerases with the high processivity required to duplicate an entire genome. Moreover, PCNA acts as a scaffold protein to facilitate recruitment of proteins to replication fork (Moldovan et al., 2007). In addition to DNA replication, PCNA plays also important roles in multiple DNA repair pathways (Maga and Hubscher, 2003). In eukaryotes PCNA is required for efficient DNA synthesis by Pol δ or Pol ε in LP-BER (Stucki et al., 1998) and also in SP-BER by interacting with Pol β and XRCC1 (Kedar et al., 2002; Fan et al., 2004). Interestingly, PCNA appears to be involved not only in the DNA synthesis step, since it interacts with multiple BER factors acting in other BER stages, such as UNG, MPG, MUTYH, NTHL1, APE1, APE2, FEN1, and Lig I (Maga and Hubscher, 2003).

Eukaryotic genomes possess at least one gene copy encoding PCNA. In mice and humans, one PCNA gene and several pseudogenes are present (Almendral et al., 1987; Ku et al., 1989; Travali et al., 1989; Yamaguchi et al., 1991). Plants such as *Oryza sativa* (rice) or *Pisum sativa* also contain a single-copy PCNA gene, but other species like *Arabidopsis* or *Zea mays* possess at least two PCNA paralogs (Lopez et al., 1997; Shultz et al., 2007; Strzalka and Ziemienowicz, 2011).

The *Arabidopsis* genome encodes two nearly identical PCNA genes. The AtPCNA1 (AT1G07370) and AtPCNA2 (AT2G29570) proteins have been purified and crystallized, and it has been demonstrated that they conserve a three-dimensional structure very similar to that of human PCNA (Strzalka et al., 2009). AtPCNA2 interacts with AtPolλ and enhances its bypass activity on oxidative DNA damage (Amoroso et al., 2011). However, no data have been yet reported on the involvement of plant PCNA homologs in BER.

### The Scaffolding Protein X-Ray Cross-Complementation Group 1 (XRCC1)

XRCC1 does not exhibit any enzymatic activity but plays a major role in BER and SSBR pathways interacting with multiple components and facilitating repair (Caldecott, 2003). As mentioned above, mammalian XRCC1 functions in SP-BER (Cappelli et al., 1997) interacting with LigIIIα and enhancing its DNA ligase activity (Caldecott et al., 1994; Nash et al., 1997) In mammalian cells, additional interaction partners of XRCC1 in BER have been described, such as hOGG1 (Marsin et al., 2003), UNG2 (Akbari et al., 2010), hNEIL1 (Wiederhold et al., 2004), hNEIL2, MPG, hNTH1 (Campalans et al., 2005), PNKP (Whitehouse et al., 2001), APE1 (Vidal et al., 2001), or DNA Pol β (Kubota et al., 1996). Mammalian XRCC1 proteins possess two BRCT (BRCA1 C-terminal) domains (BRCT1 and BRCT2) implicated in protein–protein interactions between XRCC1 and poly (ADP-ribose) polymerase (PARP) proteins and DNA Ligase IIIα, respectively (Hanssen-Bauer et al., 2012). Interaction of XRCC1 through its BRCT2 domain with DNA Lig IIIα stimulates its DNA ligation activity (Caldecott et al., 1994; Nash et al., 1997).XRCC1 knock-out mice are embryonic lethal and show increase DNA breakage (Tebbs et al., 1999; Thompson and West, 2000). In contrast, XRCC1 deficiency has no drastic consequences in plants. The *Arabidopsis* genome encodes an XRCC1 ortholog (AT1G80420) (Taylor et al., 2002), and plant *xrcc1* mutants develop normally, although they show radiosensitivity (Charbonnel et al., 2010). Rice OsXRCC1 interacts with ss- and ds-DNA, as well as with OsPCNA *in vivo* and *in vitro* (Uchiyama et al., 2008). The *Arabidopsis* XRCC1 protein stimulates uracil BER *in vitro* (Cordoba-Cañero et al., 2009) and is required for efficient DNA ligation, probably through interaction with AtLIG1 (Martinez-Macias et al., 2013). In agreement with the absence of a DNA ligase III homolog, plant XRCC1 lacks a BRCT2 domain (Taylor et al., 2002; Uchiyama et al., 2008). *Arabidopsis* XRCC1 also stimulates the 3′-DNA phosphatase activity of ZDP (Martinez-Macias et al., 2013).

### Nick Sensors: Poly (ADP-Ribose) Polymerases (PARP)

Another type of proteins involved in the recruitment of BER enzymes are poly (ADP-ribose) polymerases (PARP). These proteins detect and bind tightly DNA strand breaks, signaling recruitment of repair proteins to the damaged site (Caldecott et al., 1996). The mammalian PARP family includes 17 proteins with homology to PARP1, its founding member (Schreiber et al., 2006; Hassa and Hottiger, 2008). In response to damage, PARP1 binds DNA strand breaks and is thereby activated to catalyze the synthesis of poly ADP-ribose (PAR) by transferring ADP-ribose from NAD^+^ to both itself and nuclear target proteins (Schreiber et al., 2006). Mammalian PARP1 is the most extensively studied PARP protein, and evidences of its role in BER have accumulated. The participation of PARP1 in BER has been demonstrated in association with XRCC1 (Caldecott et al., 1996; Masson et al., 1998), and the requirement of PARP1 in both SP and LP-BER has been reported (Dantzer et al., 1999; Dantzer et al., 2000). Additionally, it has been found that PARP2 interacts with XRCC1 and belongs to a BER complex containing XRCC1, PARP1, DNA Pol β, and DNA LigIII (Schreiber et al., 2002). Both PARP1- and PARP2-deficient cells display a significant delay in resealing of DNA strand breaks (Trucco et al., 1998; Beneke et al., 2000; Schreiber et al., 2002). However, *in vitro* repair reactions using PARP1-deficient mice extracts showed to be partially compromised (Allinson et al., 2003), and since the pathway can be reconstituted with purified enzymes in the absence of PARP, it has been suggested that this protein is dispensable for BER, at least *in vitro*.

In contrast to mammals, the *Arabidopsis* genome contains only three genes encoding PARPs: AtPARP1 (AT2Gg31320), AtPARP2 (AT4G02390), and AtPARP3 (AT5G22470), with homology to human PARP1, PARP2, and PARP3, respectively (Babiychuk et al., 1998; Rissel et al., 2014; Vainonen et al., 2016). AtPARP1 and AtPARP2 seem to be broadly expressed, whereas AtPARP3 is detected mostly in developing seeds (Becerra et al., 2006). AtPARP1 and AtPARP2 localize to the nucleus and possess poly (ADP-ribose) polymerase activity, although AtPARP2 shows higher levels of activity than AtPARP1 (Feng et al., 2015). It has been suggested that variant residues at the active site in AtPARP3 could eliminate NAD^+^ binding and, therefore, enzymatic activity (Lamb et al., 2012). Like in animals, plant PARPs play a role in DNA repair processes. In *Arabidopsis*, increasing levels of PARP expression after DNA damage have been described (Doucet-Chabeaud et al., 2001; Waterworth et al., 2010; Dubois et al., 2011), although it has been suggested that AtPARP2 plays the major role in response to ionizing radiation (Song et al., 2015). *Arabidopsis* single *atparp* null mutants are viable and, in contrast to animals, *atparp1 atparp2* double mutants are also viable (Boltz et al., 2014). Single mutant *atparp2* plants are more sensitive to DNA damaging agents than wild-type or *atparp1* plants (Song et al., 2015), whereas double *atparp1 atparp2* mutants exhibited further increased sensitivity (Boltz et al., 2014). A role of AtPARP3 in the repair of DNA damage accumulated during seed storage has also been suggested (Rissel et al., 2014). However, a function for plant PARP enzymes in BER has not yet been stablished.

## Open Questions and Future Challenges

Significant advances have been achieved in the biochemical and genetic analysis of plant BER. However, much remains to be elucidated regarding several important issues. A major unresolved question is the identity of the DNA polymerase(s) involved in gap filling. Although several indirect lines of evidence point to Pol λ, direct proof of its involvement in plant BER is still lacking, and the possible role of other DNA polymerases cannot be ruled out. An additional important area to be explored is the deployment of BER factors in a chromatin environment. Plant BER has been successfully studied *in vitro* with purified proteins or cell extracts using naked DNA substrates, but identification of additional BER factors will certainly require more complex approaches using nucleosome substrates. The interaction between BER proteins and factors that facilitate DNA accessibility in chromatin is likely to play an important role in BER efficiency and may dictate the spatial distribution of endogenous and exogenous DNA damage across the plant genome. It will also be important to clarify whether specific BER pathways operate in plant mitochondria and/or chloroplasts, as well as to identify the main proteins involved. As with BER studies in other organisms, advances in addressing these and other challenges could be accelerated by the development of novel BER assays with *in vivo*, rather than *in vitro*, endpoints. Additionally, increased BER knowledge will undoubtedly have an impact in the emerging field of CRISPR/Cas-mediated precision genome editing, which holds enormous potential for plant breeding and crop improvement (Puchta, 2017). For example, targeted C:G-to-T:A base pair substitution can be achieved by expressing dCas9–cytidine deaminase fusions, but lower than expected conversion efficiencies have been detected (Komor et al., 2016; Nishida et al., 2016). However, additional co-expression of the specific UDG inhibitor Ugi partially inhibited endogenous BER of U:G intermediates, leading to increased levels of base substitution (Komor et al., 2016; Nishida et al., 2016). In summary, it is most likely that the near future will bring new and exciting results on this critical DNA repair pathway and its physiological roles in plants, as well as promising applications in existing and upcoming DNA technologies.

## Author Contributions

TR-A, RA, and DC-C jointly wrote the manuscript.

## Funding

Funding was provided by the Spanish Ministry of Science, Innovation and Universities, as well as the European Regional Development Fund, under Grant BFU2016-80728-P.

## Conflict of Interest Statement

The authors declare that the research was conducted in the absence of any commercial or financial relationships that could be construed as a potential conflict of interest.
